# Intramedullary Endometriosis of the Conus Medullaris

**DOI:** 10.7759/cureus.14581

**Published:** 2021-04-20

**Authors:** Theodoro B Beck, Mauren Beatriz F Carbonar, Ricardo Hanel, Ricardo T Beck, Francisco Carbonar

**Affiliations:** 1 Surgery, Maternidade Curitiba, Curitiba, BRA; 2 Internal Medicine, Maternidade Curitiba, Curitiba, BRA; 3 Neurological Surgery, Baptist Neurological Institute lyerly Neurosurgery, Jacksonville, USA; 4 Human Reproduction, Maternidade Curitiba, Curitiba, BRA; 5 Surgery, Universidade Pontifical Catholic University of Paraná, Curitiba, BRA

**Keywords:** endometriosis, conus medullaris, paresthesia

## Abstract

Endometriosis (EM) is a common gynecological disease characterized by endometrial-like tissue outside the uterine cavity. We report a case of intramedullary EM, a rare condition with only seven similar cases reported until today. MRI showed a mass-like lesion within the spinal canal at the L1-L2 levels and the histological and immunohistochemical features were characteristic of intraspinal endometriosis (IEM). A review of the relevant literature and a comparison between our case and seven other similar cases were made. Intraspinal EM must be recognized as a potential cause of periodic neurological signs and symptoms in young and middle-aged women. Timely intervention and appropriate management can result in control of the disease and an improvement in neurological functions.

## Introduction

Endometriosis (EM) is one of the most common gynecological diseases needing treatment [1]. The disease is characterized by the presence of glands and stroma (endometrial-like tissue) outside the uterine cavity [2]. It is mainly reported within the pelvic cavity, primarily on the pelvic peritoneum, ovaries, and rectovaginal septum, and in rare cases on the diaphragm, pleura, and pericardium [3]. Intraspinal EM (IEM) is rare and has only been reported a few times in the literature [4]. In the present article, we report a case of intramedullary EM, discuss the clinical aspects, radiological and histopathological features, and review the relevant literature.

## Case presentation

**Figure 1:**
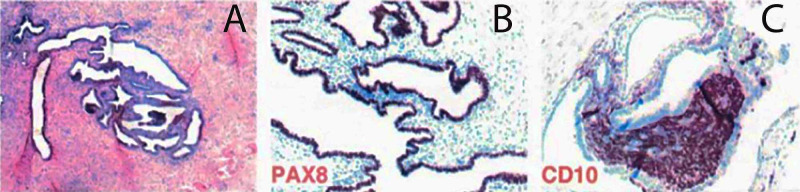
Histological section of the tumor sample Histological section showing nodular tissue composed of smooth muscle bundles and cystic glands. Histological sections demonstrate the presence of endometrial stroma and glandular epithelial tissue surrounded by fibroadipose tissue (A). PAX8 by epithelial tissue and estrogen receptors in stromal and epithelial cells (B); immunohistochemistry showed CD10 expression by stromal cells (C).

**Figure 2:**
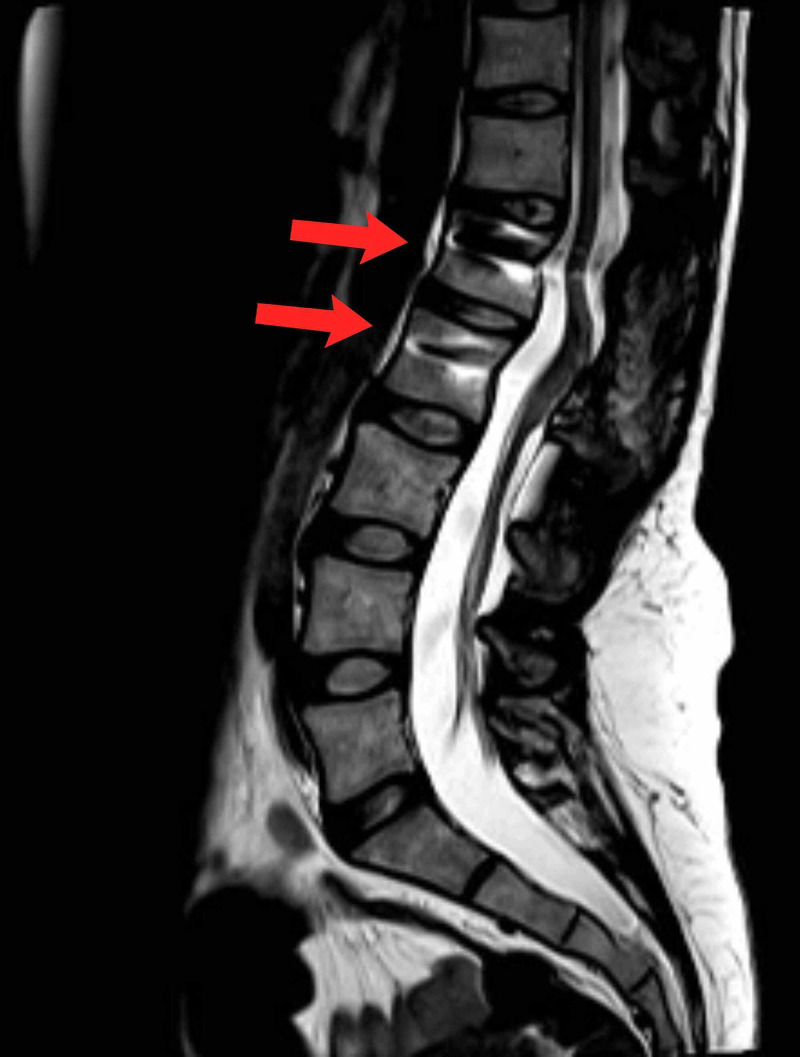
Sagital magnetic resonance imaging Postoperative T2 sagittal MRI scan at the levels L1 and L2.

A 30-year-old woman presented with sudden onset of lower limb paresthesia which awakened her at night and was associated with vertigo. The patient presented with a decrease in lower limb sensation to sharp and dull point discrimination bilaterally on physical examination. Strength was mildly weak, no urinary or intestinal symptoms were reported, and findings did not correlate with her menstrual cycles. The past morbid history described the patient as nulliparous, menarche at 12 years, regular menstrual cycles, and no related pain. Gynecological examination and abdomen ultrasound were normal. Magnetic resonance imaging (MRI) of the dorsolumbar region showed an intramedullary nodular lesion at the conus medullaris at levels L1 and L2 with a heterogeneous signal. The image was partially isotensive and hypointense in T1 and hyperintense in T2, measuring 20 × 13 × 08 mm^3^ (cephalo-caudal × transverse × ventrodorsal). Symmetrical expansion of the cord was evident, and surrounding medullary edema was also observed. Differential diagnosis included underlying neoplasia. The patient underwent surgical treatment with complete removal of the mass from the conus medullaris. The mass was firm, with an avascular appearance; no signs of previous bleeding were identified. After removal of the mass, spinal instrumentation was necessary, with no further injury to the cord and surrounding nerve roots. Gross examination of the surgical specimen revealed nodular tissue composed of smooth muscle bundles and cystic glands. Histological sections demonstrated the presence of endometrial stroma and glandular epithelial tissue surrounded by fibro adipose tissue. Immunohistochemistry showed CD10 expression by stromal cells, PAX8 by epithelial tissue, and estrogen receptors in stromal and epithelial cells (Figure 1). Complete improvement of sensory and motor deficits was achieved bilaterally. However, she remained on hormonal therapy for one year with Goserelin (Zoladex). A postoperative sagittal MRI scan showed a successful resection of the lesion (Figure 2).

## Discussion

Endometriosis is a common gynecological disorder that affects 6% to 15% of women of reproductive age [3]. Presentations are diverse, and the most common symptoms are pelvic pain, dysmenorrhea, and dyspareunia [2]. EM within the central nervous system is an infrequent condition, with only a handful of cases reported in the spinal cord [4]. Most cases of IEM presented a mass with hematoma. Thus, the differential diagnosis included underlying neoplasm or vascular lesion [4-7]. However, our patient had a spinal mass with no evidence of bleeding, and neurologic symptoms had no relation to her menstrual cycles, a presentation that makes this case report unique. The rarity of the disease and lack of specific symptoms increase the difficulty of identifying the spinal mass origin [5,6]. The underlying cause of intramedullary EM of the spinal cord is unknown, although several hypotheses exist [7]. Proposed origins of EM are regurgitation through the fallopian tube, extrapelvic dissemination through pelvic veins, lymphatic dissemination, and metaplastic differentiation of coelomic epithelium. Anatomically, IEM probably results from the reverse transport of endometriotic tissue via Betson’s venous plexus [4].

Seven articles describing similar spinal EM cases were previously published. All cases describe female patients with an average age of 31 years, the youngest being 25 years old and the eldest 42 years old. Most presentations are described as chronic development of neurological symptoms associated with the menstrual cycle. Most symptoms were reported as lower extremity radiculopathies, and two of the cases also reported difficulty voiding. Six cases reported EM located at the conus medullaris and only one within the vertebral region. In all cases, the intramedullary nature of the lesion was initially detected by MRI imaging. The histopathology that was confirmed after surgical biopsies was obtained, except for one case published in 1968, where MRI imaging was not available [8]. Patients with lesions located at the conus medullaris were submitted to laminectomy. However, only half were furtherly treated with medically induced menopause post-operatively. The totality of cases reported improvement in their neurological symptoms, and most had remission of radicular symptoms. The uniqueness of our case resides in the fact that symptoms started with sudden onset of lower limb paresthesia, and symptoms were not associated with menstrual periods, characteristics that increased the difficulty in making the diagnosis. Another distinguishing characteristic was the absence of hematoma surrounding the lesion in the MRI. While other cases considered vascular lesions as a differential diagnosis, the atypical presentation made neoplasia the primary differential diagnosis.

Management of intramedullary spinal EM involves both medical and surgical therapies [7]. Some articles that report similar spinal EM suggest that attempts at total removal of spinal cord EM may be safer after achieving pharmacological control [7]. Our patient etiological diagnosis was only possible after the removal of the tumor and histological analysis. Therefore, surgery was performed without prior pharmacological treatment.

IEM is a rare manifestation of endometriosis, and management is not well established by the literature. Drug therapy can be the initial treatment choice if IEM is suspected [9]. Patients who present with occupancy of the nidus in the spinal canal might suffer from spinal cord- or cauda equina-related deficits. Patients who do not respond to drug therapy or with frequent recurrence can be managed surgically [9]. Total removal of the spinal cord IEM might be safer after achieving pharmacological control [7]. Drug therapy post-surgery is also an option and should be initiated as soon as confirmation of the diagnosis is made [7].

## Conclusions

This rare case of IEM demonstrates the importance of maintaining a broad differential diagnosis when evaluating spinal cord injuries and the necessity of a comprehensive history for each patient. Young female patients with acute or menstruation-related neurological symptoms should raise suspicion for IEM. Most IEM reported cases are associated with an actively bleeding mass. However, a mass intraspinal lesion without evident hematoma must also include EM as a differential diagnosis. Moreover, timely intervention and appropriate management in patients with neurological symptoms can control the disease and improve neurological function.
